# Deficiency of the Transcriptional Repressor B Cell Lymphoma 6 (Bcl6) Is Accompanied by Dysregulated Lipid Metabolism

**DOI:** 10.1371/journal.pone.0097090

**Published:** 2014-06-03

**Authors:** Christopher R. LaPensee, Grace Lin, Alexander L. Dent, Jessica Schwartz

**Affiliations:** 1 Department of Molecular & Integrative Physiology, Univ Michigan, Ann Arbor, Michigan, United States of America; 2 Graduate Program in Cellular & Molecular Biology, Univ Michigan, Ann Arbor, Michigan, United States of America; 3 Dept of Microbiology & Immunology, Indiana Univ School of Medicine, Indianapolis, Indiana, United States of America; University of Minnesota - Twin Cities, United States of America

## Abstract

The transcriptional repressor B-cell Lymphoma 6 (Bcl6) was recently identified in a profile of genes regulated in adipocytes, suggesting a relationship between Bcl6 and metabolic regulation. As a representative target gene repressed by Bcl6, Suppressor of Cytokine Signaling (Socs) 2 expression was elevated in Bcl6 deficient (KO) mice, including metabolic tissues liver, adipose tissue and muscle, as well as in spleen and thymus. Bcl6 occupied the Socs2 promoter in wild-type, but not Bcl6 KO mice, suggesting direct regulation of Socs2 by Bcl6 *in vivo*. Mice deficient in Bcl6 were found to exhibit multiple features of dysregulated lipid metabolism. Adipose tissue mass was dramatically reduced or absent in Bcl6 KO mice. Further, hepatic and serum triglycerides were low. Bcl6 deficiency was accompanied by decreased hepatic expression of Stearoyl-CoA desaturase 1 (Scd1) and Fatty acid synthase (Fasn) genes which encode lipogenic enzymes. Expression of the gene for the transcription factor Carbohydrate-Responsive Element Binding Protein (Chrebp), which regulates expression of lipogenic genes, was also reduced in liver of Bcl6 KO mice. Bcl6 deficiency disrupted fasting-induced increases in hepatic triglyceride deposition, but not decreases in lipogenic gene expression. Taken together, these findings suggest that in addition to its well-recognized roles in immune regulation, Bcl6 plays a role in regulatory events of lipid metabolism, and that in the absence of Bcl6, lipid metabolism in liver and adipose tissue is dysregulated.

## Introduction

Bcl6 (B-cell lymphoma 6) is a transcriptional repressor protein known for its ability to inhibit gene expression during B-cell responses in germinal centers [1]–[3]. Bcl6 prevents premature activation and differentiation of B cells, drives differentiation of T follicular helper cells, and facilitates the production of high-affinity antibodies. Chromosomal translocations in the Bcl6 gene and mutations in the Bcl6 gene promoter can cause deregulation of Bc6 expression by preventing its down-regulation in post-germinal center B cells [1], [3]–[5]. As a result, Bcl6 can act as an oncogene in germinal center-derived lymphomas such as Diffuse Large B-cell Lymphoma [1], [3]–[5].

Bcl6 expression is not restricted to immune tissues however, and Bcl6 has been reported to participate in many physiological processes, such as repressing inflammation [6]–[8], protecting testicular germ cells from apoptosis [9], repressing proliferation of pancreatic β cells [10], and mediating sexual dimorphism of gene expression in the liver [11], [12]. Bcl6 was recently found to be expressed and inhibited in adipocytes treated with Growth Hormone (GH) [13], raising the possibility that Bcl6 has unrecognized functions in adipocytes and may be a player in the regulation of lipid metabolism.

Bcl6 has been implicated as a repressor in mediating GH-regulated transcription. For example, Bcl6 occupies the promoter of the GH-induced gene Suppressor of Cytokine Signaling 2 (Socs2), whose product negatively regulates cytokine signaling, in adipocytes. Further, transcriptional activation analysis has shown that Bcl6 inhibits Socs2-luciferase [13], demonstrating that Bcl6 can function as a transcriptional repressor of Socs2 *in vitro*. These observations suggest potential roles for Bcl6 in regulating genes associated with metabolic regulation, particularly lipid metabolism.

The regulation of lipid metabolism is largely carried out by adipose tissue and liver. Adipose tissue serves as a major site of energy storage in the form of triglycerides, which are released during lipolysis as free fatty acids and glycerol for use by peripheral tissues such as muscle and liver. The liver is another crucial site of lipid metabolism, serving as a hub of fatty acid and triglyceride synthesis, cholesterol biosynthesis and lipid distribution into the circulation. The triglyceride content of liver reflects a balance in the relative rates of hepatic fatty acid lipogenesis, oxidation, uptake from circulation, and output as very low density lipoproteins. Fatty acid synthesis and oxidation are under the control of enzymes whose gene expression is regulated by multiple transcription factors [14]–[17]. For example, expression of the genes for the lipogenic enzymes Fatty Acid Synthase (FAS), which catalyzes formation of fatty acids from acetyl-CoA and malonyl-CoA, as well as Stearoyl Co-A Desaturase 1 (SCD1) which catalyzes the rate-limiting step in the biosynthesis of mono-unsaturated fatty acids used to synthesize triglycerides, is dependent on the activity and expression of the transcription factors Sterol Response Element-Binding Protein (SREBP) 1c and Carbohydrate-Responsive Element Binding Protein (ChREBP) [18]–[20]. With respect to fatty acid oxidation in liver, Acyl-CoA Oxidase (Acox) and Carnitine Palmitoyltransferase (Cpt1) genes, which code for enzymes that catalyze the rate-determining steps in peroxisomal and mitochondrial oxidation respectively, are under the control of Peroxisome-Proliferator Activated Receptor (PPAR) alpha. Analysis of transcription factors such as SREBP and PPAR family members has been illuminating in identifying their gene targets and roles in lipid metabolism, particularly through studies of mice lacking their expression [14]–[17]. Here, Bcl6 deficient (KO) mice were studied for insight into relationships between Bcl6 and metabolic regulation.

In light of the established role of Bcl6 as a transcriptional repressor and also the expression and regulation of Bcl6 in adipose tissue, this study investigates whether deficiency of Bcl6 is associated with changes in lipid metabolism and expression of genes involved in its regulation. Bcl6 KO mice were examined for insight into potential roles of endogenous Bcl6 in regulating target genes in traditional metabolic tissues, including genes associated with energy metabolism. In support of the role of Bcl6 in transcriptional repression in adipose tissue, in the present study, Bcl6 deficiency was found to be associated with a dramatic increase in the expression of the gene encoding Socs2 in adipose, liver and other tissues, as well as in cells isolated from Bcl6 KO mice. Consistent with a role for Bcl6 in lipid metabolism, Bcl6 deficiency in mice was also accompanied by a marked decrease in adipose tissue mass compared to WT mice. As an additional indicator of dysregulation of lipid metabolism, hepatic and serum triglyceride levels were reduced in Bcl6 KO mice. The expression of key lipogenic genes and the transcription factor ChREBP, which can regulate them, was lower in the liver of Bcl6 KO mice than in WT mice. Further, a fasting-induced increase in hepatic triglyceride content was impaired in Bcl6 KO mice. Overall, the absence of Bcl6 in mice results in dysregulation of lipid metabolism, characterized by suppression of lipogenic pathways and decreased lipid deposition, identifying a new dimension in the roles of endogenous Bcl6.

## Materials and Methods

### Bcl6-deficient mice

The generation of mice with targeted deficiency of Bcl6 has been described previously [8]. In some of the present studies, tissues were obtained from a colony of Bcl6 KO mice housed at the Indiana University School of Medicine. Bcl6 heterozygous (+/−) male and female mice from the Indiana colony were used to establish a breeding colony at the University of Michigan to generate Bcl6 KO mice. As reported previously, Bcl6 KO mice show poor survival and rarely live past 5–6 weeks of age [8]. All parameters tested were comparable in tissues from mice from Indiana and Michigan colonies. Pups were genotyped by PCR analysis of tail DNA as previously described [21]. Mice were housed on a 12-h light, 12-h dark cycle (lights on at 0600 h), with food and water available *ad libitum* unless otherwise specified. Male and female mice 5–7 wk old were euthanized between 1000 and 1200 h by CO_2_ inhalation and cervical dislocation. Blood was obtained by cardiac puncture, clotted and centrifuged at 5000× *g* for 10 min at 4 C for collection of serum, which was stored at −80 C for later analysis. Epidydimal adipose tissue, liver and other tissues were rapidly removed, frozen on dry ice and stored at −80C. For fasting experiments, mice were placed in a clean cage with water but no food for 16 hr. Animal protocols were approved by the Indiana University School of Medicine Animal Use and Care Committee and the University Committee on Use and Care of Animals at the University of Michigan.

### Cell Culture

Primary cultures of mesenchymal stem cells (MSCs) were prepared from ears of 5-week-old mice and subjected to both mechanical and enzymatic dissociation as previously described [22]. Briefly, ears were minced and digested with collagenase type I (Worthington Biochemical, Freehold, NJ) for 1 h at 37°C with gentle agitation. The cell suspension was filtered through a 70-µm cell strainer (Becton Dickinson Labware, Franklin Lakes, NJ), centrifuged, and resuspended for 1 min in 1 mL per mouse of red blood cell lysis buffer (Sigma Co., St. Louis, MO). The isolated cells were plated in 12 well plates at 80,000 cells/well in Dulbecco's modified Eagle medium/F12 (DMEM/F12; Invitrogen, Carlsbad, CA) supplemented with 1% Primocin and 15% FBS, and were used when confluent.

### Materials

TRIzol Reagent was purchased from Invitrogen, and Taqman Reverse Transcription Kit from Applied Biosystems (Carlsbad, CA). Protease inhibitors leupeptin and aprotinin were purchased from Roche (Indianapolis, IN), and phenylmethylsulfonylfluoride (PMSF) from Mallinckrodt (St Louis, MO). Sodium orthovanadate, SYBR green, and Serum Triglyceride Determination Kit were purchased from Sigma (St. Louis, MO). Nitrocellulose membrane was purchased from GE Life Sciences (Piscataway, NJ). Protein molecular weight standards were purchased from Invitrogen (Grand Island, NY) and BioRad (Hercules, CA). Primocin was purchased from Invivogen (San Diego, CA).

### Quantitative Real-time PCR (qpcr)

qpcr was performed on total RNA isolated from tissues and analyzed using the 2^ΔΔcT^ method [23]. The sequences of qpcr primers are based on published studies or were designed using NCBI Primer Tool (Table S1 in File S1). Gene expression was normalized to RPLP0 (Ribosomal protein, large P0), and is expressed as fold-change compared to control group, where the control is normalized to 1 unless otherwise indicated.

### Chromatin Immunoprecipitation (ChIP)

ChIP was carried out as described [24] with the following modifications for analysis of liver: Frozen mouse liver (∼200 mg) was thawed, minced in 3 ml PBS and incubated in 1% formaldehyde in PBS for 15 min at room temperature before centrifugation for 2 min at 1500 rpm. The pellet was suspended in ChIP SDS lysis buffer [24] and sonicated eighteen times for 15 seconds, with a 1 minute pause between cycles, to achieve approximately 200 bp fragments. For each immunoprecipitation, 100 µg of liver protein was incubated overnight at 4 C with 4 µg of anti-Bcl6 (N-3, Santa Cruz). Samples incubated with equivalent amounts of normal rabbit IgG served as negative controls. 1% input was used to indicate the relative amount of each sample used for individual ChIP analysis. ChIP samples were analyzed by PCR using primers targeting the Bcl6 binding site in the murine Socs2 gene [13]. PCR products were separated on 2% agarose gels and stained with ethidium bromide.

### Immunoblotting analysis

Aliquots of liver (∼200 mg) were thawed and homogenized 15 times in a Dounce homogenizer in 1 ml ice-cold homogenization buffer [25]. Cellular debris was cleared by centrifuging (13,000 rpm) at 4°C for 10 min. Protein concentration of the supernatant was determined by Bio-Rad protein assay reagent using a BSA standard curve. For immunoblotting, 50 µg liver protein was separated on 10% SDS-polyacrylamide gels and transferred to nitrocellulose membrane. Antibodies against SOCS2 were purchased from Millipore; in liver, this antibody recognized non-specific as well as specific bands under the conditions of these experiments, as specified in figure legend. Antibodies against FAS (H-300) and SCD1 (S-15) were purchased from Santa Cruz Biotechnology, Inc. (Santa Cruz, CA). α-tubulin antibody was obtained from Cell Signaling (Danvers, MA). The secondary antibodies (1∶10000) conjugated with IRDye800 and IRDye700 were obtained from Rockland Inc (Gilbertsville, PA) and Invitrogen (Grand Island, NY) respectively. Immunoblotting was performed at 4°C overnight as described [26] with the following antibody dilutions: anti-SOCS2 (1∶1000), anti-FAS (1∶200), anti-SCD1 (1∶200), and anti-α-Tubulin (1∶1000). Proteins were visualized and bands were quantified using the Odyssey scanning system (LI-COR Biosciences). Molecular weight was estimated using MagicMark Western Standard from Invitrogen.

### Hepatic Triglyceride determination

To measure hepatic triglycerides, liver tissue (40–50 mg) was homogenized 10 times in 1.5 ml of a mixture of CHCl_3_-CH_3_OH (2∶1, v/v), followed by shaking at room temperature for 2 hr. After addition of 0.5 ml of 0.1 M NaCl, the suspension was centrifuged at 3,700 rpm for 10 min at room temperature. The lower organic phase was transferred and air-dried. The residual lipid was resuspended in 100 µl of 1% Triton X-100 in absolute ethanol, and the concentrations of triglycerides in 5 µl were determined in duplicate using the Triglyceride Colorimetric Assay Kit (Cayman Chemical, Ann Arbor MI).

### Serum metabolites

Serum from mice was pooled as described in Table 1 and tested for triglycerides, fatty acids, cholesterol, and high-density lipoprotein (HDL) by the Chemistry Core of the Michigan Diabetes Research and Training Center (http://www.med.umich.edu/mdrtc/cores/ChemCore/index.html) at the University of Michigan using Roche Cobas Mira Plus (Roche Diagnostics, Indianapolis IN). For some experiments, serum triglycerides were individually measured as described in the figure legends using the Triglyceride Colorimetric Assay Kit (Cayman Chemical, Ann Arbor MI). Tail blood was directly analyzed for glucose using a One Touch glucometer (Lifescan, Milpitas, CA).

**Table 1:** Serum metabolite profile of Bcl6 deficient and WT mice.

A. Fed/Non-fasted mice		
Serum parameter	WT	KO
Triglycerides (mg/dl)	172±20	85±9**
Free Fatty Acids (mEq/L)	1.03±0.05	0.87±0.09
Cholesterol (mg/dl)	131±5	158±17
HDL (mg/dl)	111±2	114±17
Glucose (mg/dl)	250±11	267±17

B. Fasted		
Serum parameter	WT	KO
Triglycerides (mg/dl)	188±20	72
Free Fatty Acids (mEq/L)	1.84±0.3	1.57
Cholesterol (mg/dl)	139±9	152
HDL (mg/dl)	112±8	97
Glucose (mg/dl)	122±5	135±11

Serum from (A) non-fasted or (B) fasted (16 hr) male mice aged 6 weeks were analyzed. For fed mice, values shown are the mean+se for 4 mice/genotype of triglycerides, free fatty acids, cholesterol and HDL. Significant differences from WT are designated by

**p<0.005.

Since serum samples from fasted KO were limited, some samples were pooled, and data were not analyzed statistically. Triglycerides, free fatty acids, cholesterol and HDL in Fasted WT serum was analyzed in 2 pools, with 3 mice per pool; values shown are the mean ± range of the pools. For fasted Bcl6 KO, serum was analyzed in 1 pool, with 3 mice per pool (total n = 3). Glucose levels are the mean ± se of individual determinations from n = 5 fed WT, 3 fed Bcl6 KO, 6 fasted WT, 4 fasted Bcl6 KO. Glucose levels in fasted Bcl6 KO or WT were significantly (p<0.05) lower than in fed mice of the same genotype, but glucose values were not different between KO and WT in each nutritional state.

### Statistics

Values from analysis of WT and Bcl6 KO mice were compared statistically using Student's t-test. Values from fasting/feeding experiments were evaluated by ANOVA followed by a Tukey's multiple comparison test using GraphPad Prism software.

## Results

### Bcl6 deficiency reverses repression of Socs2 gene expression

**Figure 1:**
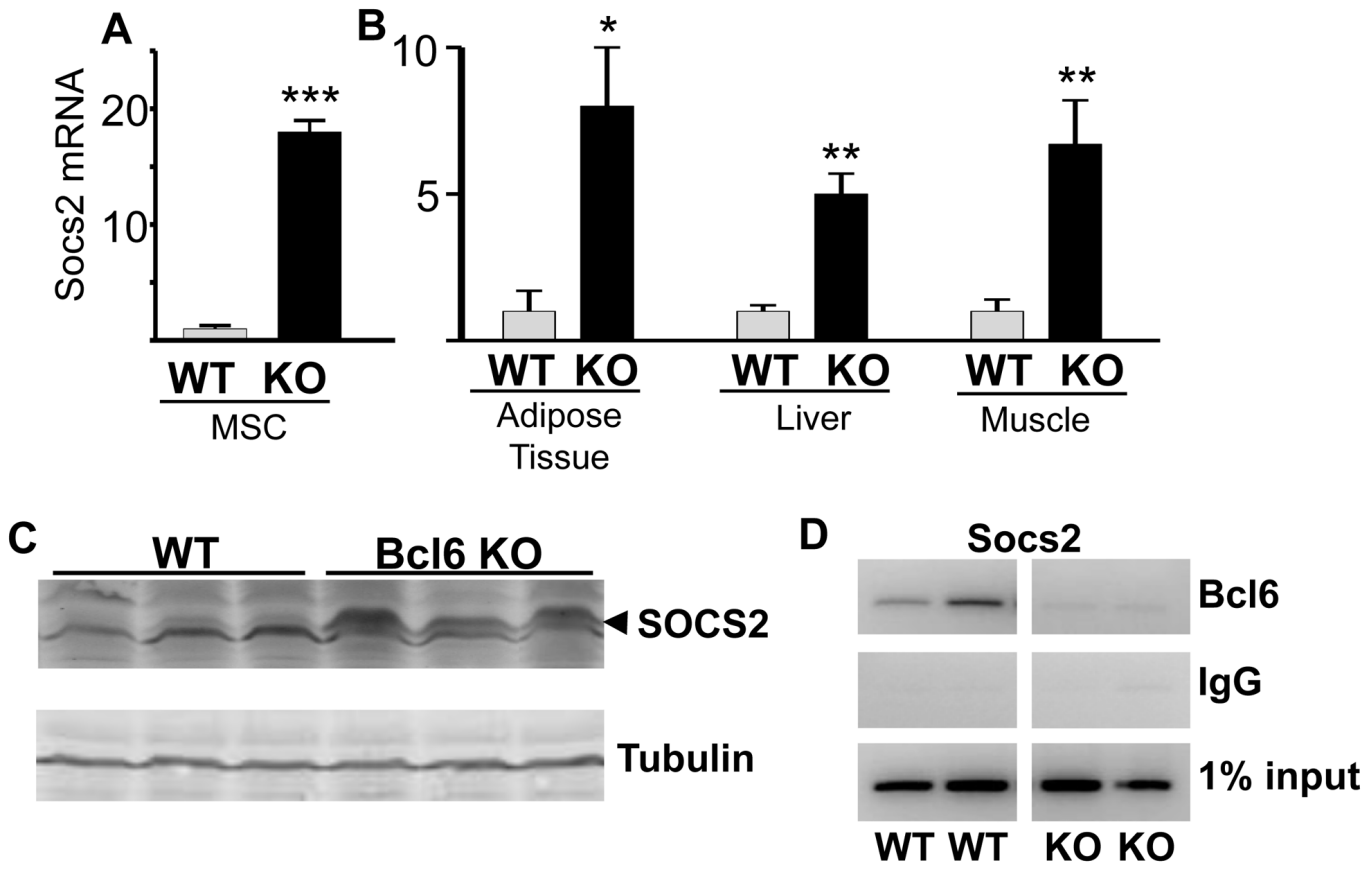
Endogenous Socs2 expression is elevated in cells and tissues from Bcl6-deficient mice. **A**- Mesenchymal stem cells (MSC) were isolated from the ears of WT and Bcl6 KO male mice and analyzed by qpcr. Bars show the mean+SE of triplicate cell preparations from a mouse of each genotype; similar results were obtained in two independent experiments. **B** - RNA isolated from adipose tissue, liver, and muscle of WT and Bcl6 KO male mice was analyzed using qpcr. mRNA expression is shown as the mean+SE for 4 mice of each genotype. Bcl6 KO are indicated by black bars and WT by grey bars, in this and subsequent figures. Significant differences from WT are designated by * (p<0.05), ** (p<0.005), and *** (p<0.0005). **C** – Lysates from livers of 3 WT and 3 KO mice were subjected to immunoblotting with anti-SOCS2. Arrowhead indicates migration of upper band as SOCS2. The lower band is non-specific. Tubulin served as a loading control. **D** - Nuclei from the livers of 2 WT and 2 Bcl6 KO mice were analyzed individually by ChIP, using antibody against Bcl6, with primers for the Bcl6-binding sequence in the Socs2 promoter [13]. IgG served as a negative control; 1% input is shown.

Deficiency of Bcl6, a transcriptional repressor, would be expected to increase expression of Bcl6 target genes. The promoter of the gene encoding SOCS2 is predicted to contain a Bcl6 consensus binding sequence (TTCCTGGAA) [13]. This sequence is occupied by endogenous Bcl6 in 3T3-F442A cells, and a reporter construct containing this sequence upstream of luciferase is inhibited by expression of Bcl6 [13]. Thus the Socs2 gene was examined as a representative Bcl6-responsive gene. Mice with a targeted deficiency of Bcl6 (Bcl6 KO) [8] were used to prepare mesenchymal stem cells (MSC) from ears. In these isolated cells deficient in endogenous Bcl6, expression of Socs2 was significantly higher than that in cells from WT mice (Fig. 1A). These findings suggest that deficiency of Bcl6 increases expression of Socs2 in a cell autonomous manner.

Consistent with the elevated Socs2 observed in Bcl6 deficient cells, Bcl6 KO mice show 6-fold higher hepatic expression of endogenous Socs2 mRNA (Fig. 1B) compared to WT mice. SOCS2 protein also appears to be more abundant in Bcl6 KO liver (Fig. 1C). Socs2 mRNA expression was also higher in adipose tissue and muscle, additional key metabolic tissues (Fig. 1B), as well as in kidney, spleen and thymus (Fig. S1 in File S1) from male Bcl6 KO compared to WT mice. Socs2 expression was elevated in the liver of female Bcl6 KO mice (Fig. S2 in File S1) to the same extent as in male Bcl6 KO mice. Chromatin immunoprecipitation (ChIP) demonstrated that the occupancy of endogenous hepatic Bcl6 on the DNA encompassing the Bcl6 binding site on the Socs2 promoter was evident only in the liver of WT mice, but was absent in Bcl6 KO mice (Fig. 1D), consistent with their deficiency of Bcl6. These observations agree with the findings of elevated Socs2 in the cells from mice deficient in Bcl6, and further support Bcl6 functioning in adipose, liver and other tissues as a transcriptional repressor of Socs2. The Bcl6-dependent changes in Socs2 expression in adipose tissue, liver and muscle suggest links between Bcl6 and metabolic regulation, which are key functions of these tissues.

### Bcl6-deficient mice exhibit reduced adipose tissue mass

**Figure 2:**
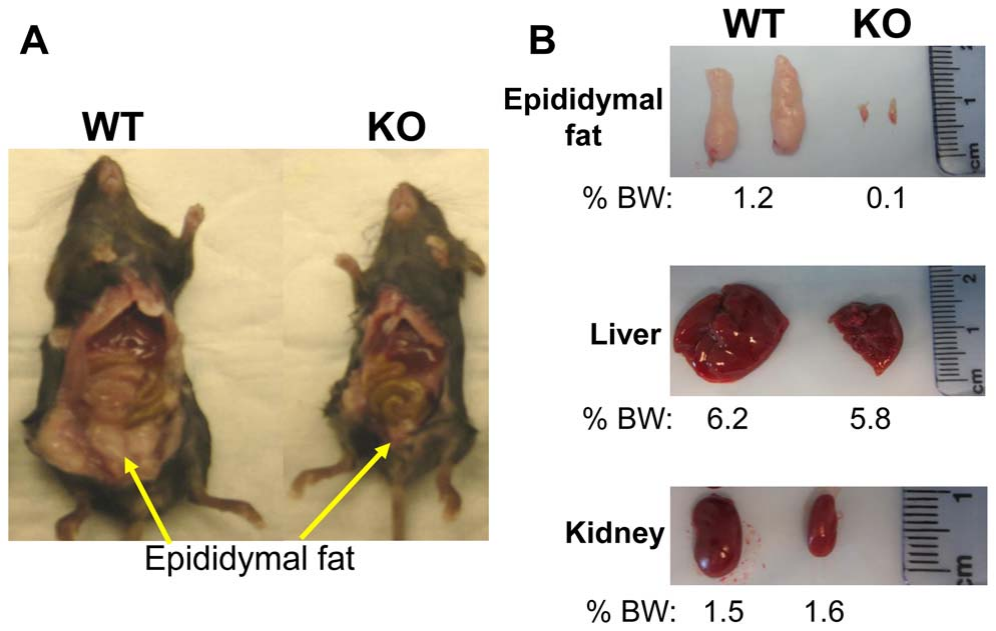
Bcl6 deficient mice are small and exhibit reduced adipose tissue mass. **A**- 6 week old male Bcl6 KO mice has reduced or undetectable epididymal adipose tissue mass (arrows) compared to WT. Similar observations were made in 100% of KO mice (n = 16). KO mice are about 40% smaller than WT (9.6±1.4 g, mean±SE KO compared to 21.7±1.0 g WT; n = 5 KO, 11 WT). **B** - Excised tissues from representative WT and KO mice are shown with tissue weight as percent BW shown below image (see text).

A dramatic phenotype in the Bcl6 KO mice is their markedly reduced adipose tissue mass (Fig. 2). Epididymal adipose tissue in Bcl6 KO mice is undetectable or disproportionately smaller relative to body weight (BW) than in WT mice, despite smaller size of KO (Fig. 2, Fig. S3 in File S1) [8]. Lack or marked decrease was also observed for periovarian fat in females (not shown). In contrast, the reduced size of other organs, including liver and kidney (Fig. 2B), lung and heart (not shown) is proportionate to the reduced size of Bcl6 KO mice (30% of WT values). For adipose tissue, the tissue weight (TW)/BW ratio in KO is less than 10% of the WT ratio, while for liver and kidney, TW/BW ratios are almost identical in KO and WT, demonstrating the disproportionate deficit of adipose tissue in the Bcl6 KO relative to the WT. The disproportionately limited amount or absence of adipose tissue mass was observed in 100% of the Bcl6 KO mice studied. These observations are suggestive that endogenous Bcl6 expression contributes to regulating genes that participate in adipocyte functions and/or lipid metabolism.

### Bcl6 KO mice exhibit impaired lipogenesis

**Figure 3:**
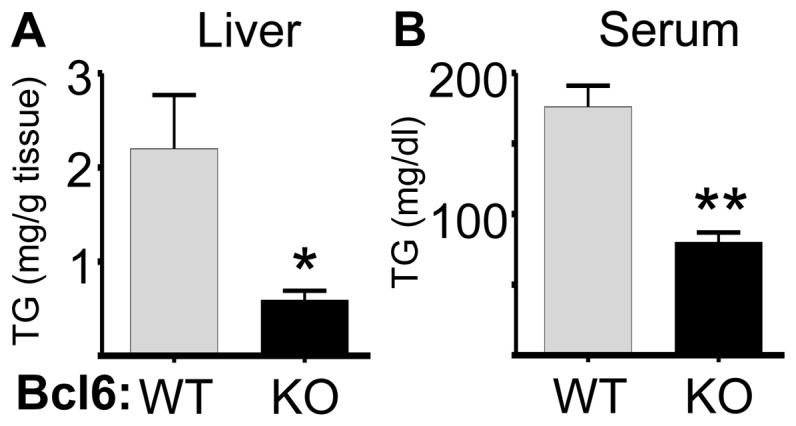
Bcl6-deficient mice exhibit reduced triglycerides. **A**- Triglycerides (TG) were measured (mg/gm tissue) in the livers of 6 week old male Bcl6 KO and WT mice as described. **B** - Serum triglycerides from the same male mice were measured as described. Values shown (mg/dl) are the mean+SE for 4 mice/genotype. Significant differences from WT are designated by * (p<0.05), and ** (p<0.005).

Since adipose tissue is scarce or absent in Bcl6 KO mice, liver was analyzed as a central organ in overall lipid metabolism for insight into a potential role of Bcl6 in triglyceride metabolism. Hepatic storage of lipid as triglyceride was found to be 70% lower in Bcl6 KO mice compared to WT littermates (Fig. 3A), indicative of dysregulated hepatic triglyceride metabolism in the absence of Bcl6. The decrease in triglyceride was observed in female (Fig. S4 in File S1) as well as male Bcl6 KO mice. Serum triglycerides were also decreased by 40–50% in Bcl6 KO mice (Fig. 3B and Table 1).

**Figure 4:**
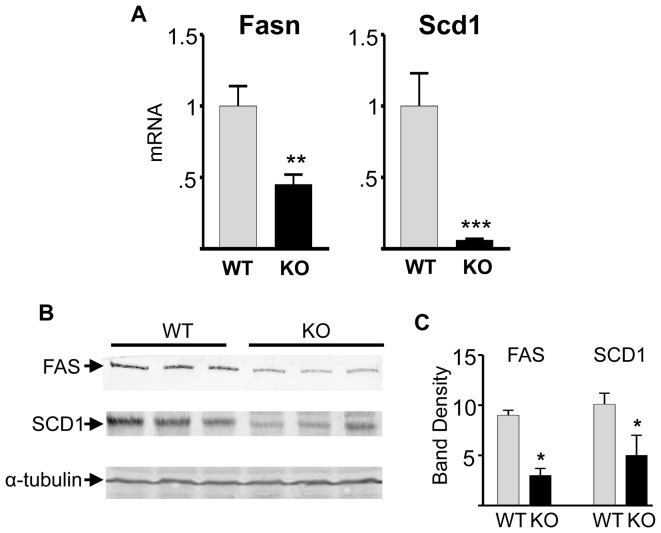
Bcl6 deficiency decreases expression of lipogenic genes and proteins in liver. **A**- Hepatic RNA was extracted from the liver of Bcl6 KO or WT mice, and expression of Fasn and Scd1 was measured by qpcr. Gene expression was calculated as fold change relative to WT mice. Bars show the mean+SE for 5 Bcl6 KO and 9 WT mice. **B** - Liver lysates were analyzed by immunoblotting using antibodies against FAS and SCD1 protein individually for 3 Bcl6 KO or 3 WT mice. α-tubulin served as a loading control. **C** - Quantification of immunoblots for FAS and SCD1 in B, normalized to α-tubulin, shown as mean+SE. Significant differences are designated by * (p<0.05), ** (p<0.005) and *** (p<0.0005).

**Figure 5:**
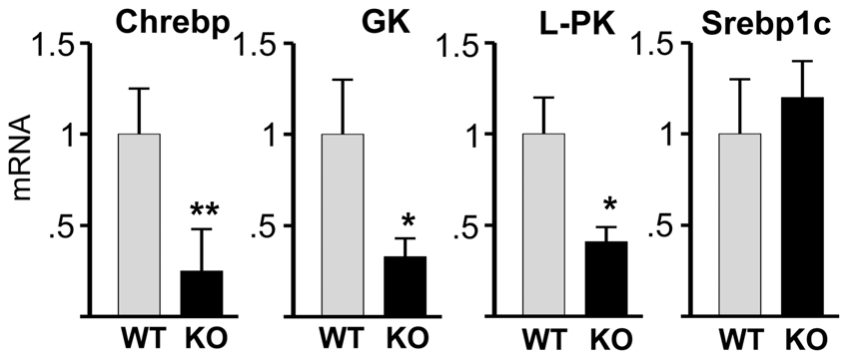
Bcl6 deficiency decreases expression of Chrebp in liver. mRNA from liver of Bcl6 KO and WT mice was analyzed by qpcr for expression of Chrebp, GK, L-PK, and Srebp1c mRNA. Bars show the mean+SE for 4 Bcl6 KO and 4 WT mice. Significant decreases in Bcl6 KO relative to WT are designated by * (p<0.05) and ** (p<0.005).

Consistent with low hepatic triglycerides, expression of the lipogenic enzyme FAS was markedly reduced at both the Fasn mRNA (Fig. 4A) and FAS protein levels (Fig. 4B and 4C) in the livers of Bcl6 KO mice compared to WT littermates. Bcl6 KO mice also exhibit a marked reduction in Scd1 mRNA and SCD1 protein expression (Fig. 4). The expression of Fasn and Scd1 genes is regulated by the transcription factors ChREBP and SREBP1c [18]–[20]. Chrebp mRNA expression was 75% lower in the liver of Bcl6 KO compared to WT mice (Fig. 5), consistent with the decreased expression of Scd1 and Fasn. ChREBP activates transcription of lipogenic genes in a manner that is dependent on the presence of the enzyme glucokinase (GK), which phosphorylates intracellular glucose [27]. Bcl6 KO mice were also found to exhibit reduced expression of GK in the liver (Fig. 5), which may contribute to reduced activity of ChREBP and expression of lipogenic genes. Bcl6 KO mice also exhibit reduced expression of another ChREBP dependent gene, L-pyruvate kinase (L-PK) (Fig. 5), consistent with studies in which ChREBP was essential for L-PK gene transcription in liver [27], [28]. Interestingly, Srebp1c mRNA was comparable in WT and KO liver (Fig. 5), suggesting that Bcl6 deficiency impairs lipogenesis independently of changes in Srebp1c gene expression. Thus, expression of the two key lipogenic genes tested is impaired in the liver of Bcl6 KO mice, and Chrebp mRNA, which encodes a transcription factor that can coordinate the expression of lipogenic genes, is also reduced in Bcl6 KO mice.

**Figure 6:**
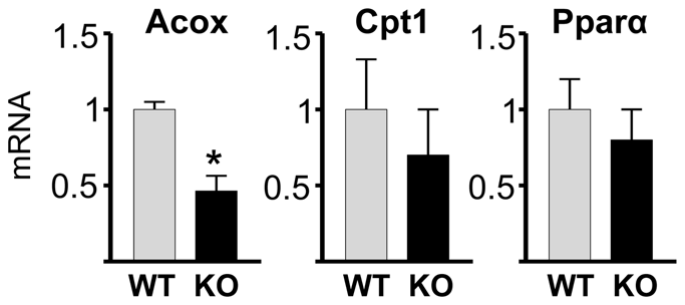
Expression of genes for fatty acid oxidation. mRNA from liver of Bcl6 KO and WT mice was analyzed by qpcr for expression of Acox, Cpt1, and Pparα. Bars show the mean+SE for 4 mice of each genotype. Gene expression was calculated as the fold-change relative to WT mice. Asterisk (*) designates significant (p<0.05) decrease in Bcl6 KO.

Since triglyceride content in the liver generally reflects a balance between synthesis and oxidation, one might expect that decreased triglycerides in Bcl6 KO mice would reflect not only impaired lipogenesis, but also elevated fatty acid oxidation. Nevertheless, expression of Acox was reduced in the liver of Bcl6 KO mice (Fig. 6), and Cpt1 expression was not significantly different in Bcl6 KO and WT mice (Fig. 6). Further, Bcl6 deficiency did not significantly change the expression of the gene for PPARα (Fig. 6), which induces genes for fatty acid oxidation including Acox and Cpt1 [29]. The reduced expression of at least one gene associated with fatty acid oxidation in the Bcl6 KO mice suggests that Bcl6 deficiency, if anything, impairs oxidation as well as synthesis of triglycerides in liver.

### Bcl6 deficiency impairs fasting-induced triglyceride deposition in liver

**Figure 7:**
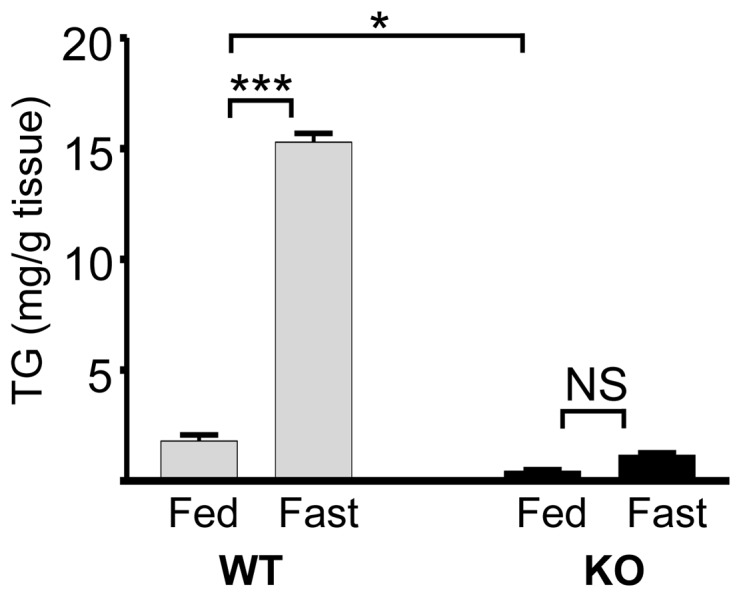
Bcl6 deficiency decreases accumulation of liver triglycerides during fasting. Triglycerides were measured in livers of fed or fasted (16 hr) Bcl6 KO and WT male mice. Bars show the mean+SE for n = 3 mice/genotype and nutritional status. Brackets indicate significant differences between bracketed bars: * (p<0.05), *** (p<0.0005), or not significant (NS).

Changes in lipid metabolism are adaptive during fasting when other energy sources are low. During a typical response to fasting in WT mice, metabolic pathways favor mobilization of triglycerides from adipose tissue to peripheral tissues, including liver. The near lack of adipose tissue in Bcl6 KO mice makes it unlikely that they respond to fasting appropriately by mobilizing lipids. When WT mice were fasted, hepatic triglyceride levels increased 8-fold (Fig. 7) [30]. In contrast, in Bcl6 KO mice, basal triglyceride levels in liver were only 20% of those in WT, and did not show a significant increase during fasting, suggesting an impairment in hepatic lipid deposition in response to fasting in Bcl6 KO mice. Serum fatty acids trended lower (15%) in Bcl6 KO than WT mice in both the fed or fasted state (Table 1B), further suggesting aberrant lipid metabolism in Bcl6 KO mice.

While the impaired elevation in triglycerides in Bcl6 KO mice suggests that adaptation to fasting is impaired, other metabolic changes typically associated with fasting were not altered by Bcl6 deficiency. Among serum metabolites (Table 1), glucose fell to the same extent (50%) during fasting in both Bcl6 KO and WT mice. Free fatty acid levels tended to rise by approximately the same amount (80%) during fasting in both Bcl6 KO and WT, though only limited data are available for KO during fasting. Serum cholesterol tended to be slightly higher in KO than WT mice in both fed and fasted states, but did not appear to change appreciably with nutritional status. Serum HDL were detectable in in KO and WT mice when fed or fasted, but trends could not be evaluated.

**Figure 8:**
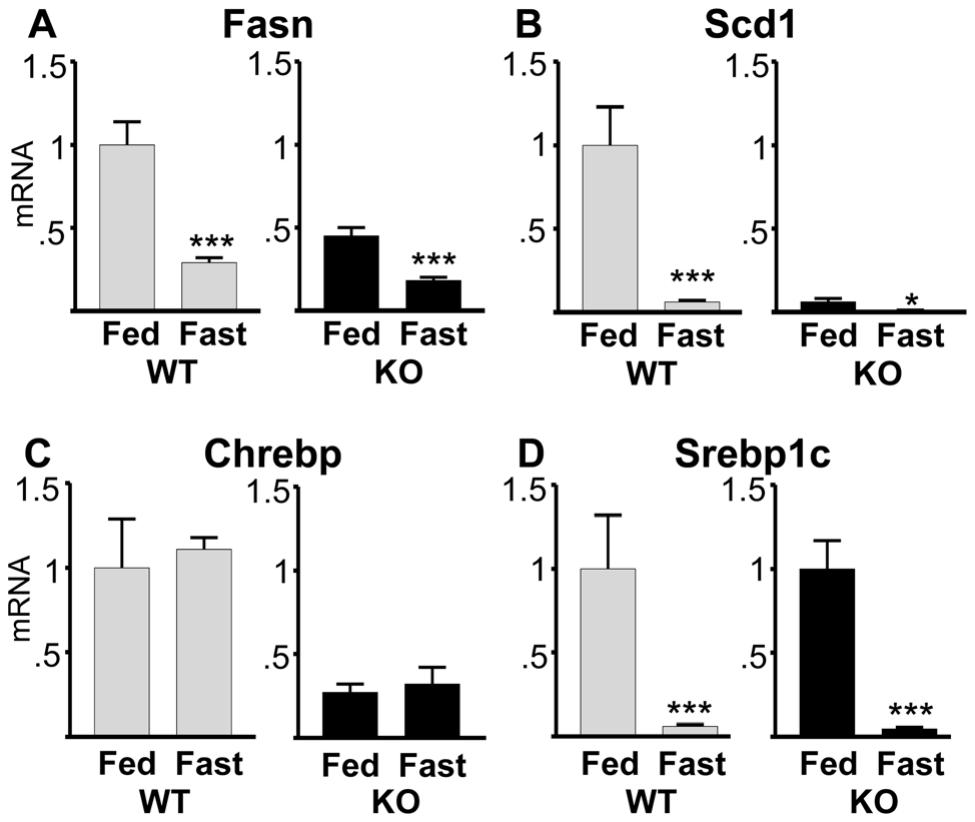
Fasting-induced decreases of lipogenic gene expression are unimpaired in Bcl6 KO mice. Bcl6 KO (black bars) and WT male mice (gray bars) were either provided with food *ad libitum* (fed) or subjected to a 16 hr fast. Expression of hepatic (**A**) Fasn, (**B**) Scd1, (**C**) Chrebp, and (**D**) Srebp1c mRNA were measured using qpcr. Gene expression in fasted mice was calculated compared to fed mice, with fed state set to 1 for fed WT. Measurements are expressed as the mean+SE for n = fed: 9 WT, 5 KO, fasted: 5 WT, and 3 KO mice. Fasting decreased Scd1 expression in KO by 78%. Significant differences from fed mice of the same genotype are designated by * (p<0.05), and *** (p<0.0005).

With respect to hepatic lipogenesis, fasting in WT mice significantly decreased expression of Fasn and Scd1 mRNA by 70% and 90% respectively (Fig. 8A and B, gray bars), as expected. In Bcl6 KO mice (black bars), fasting decreased expression of Fasn and Scd1 as in the WT. Despite already being expressed at lower basal levels in fed Bcl6 KO mice (Fig. 4A), Fasn expression decreased further during fasting, dropping by 65% (Fig. 8A), while Scd1 mRNA (Fig. 8B) decreased by 78% during fasting compared to fed KO.

Decreased hepatic triglyceride synthesis in fasting is accomplished in part through reductions in expression of lipogenic genes, mediated by changes in the activity and/or mRNA levels of Chrebp and Srebp1c [19], [20], [31], [32]. WT mice exhibit comparable levels of Chrebp expression in both the fed and fasted states (Fig. 8C). Chrebp expression in fed Bcl6 KO mice is 70% lower than in fed WT, and did not change significantly with fasting. On the other hand, Srebp1c expression was significantly reduced during fasting in the liver of both WT and Bcl6 KO mice, decreasing by about 90% in either genotype (Fig. 8D). Thus, Bcl6 deficiency did not prevent down-regulation of lipogenic genes during fasting, although it did impair triglyceride accumulation in liver.

## Discussion

### Endogenous Bcl6 deficiency increases Socs2 expression

Because Bcl6 is a transcriptional repressor, the consequence of Bcl6 deficiency can reverse repression and contribute to gene activation [33]. *In vitro* studies have established Socs2 as a Bcl6 target gene, where occupancy of endogenous Bcl6 on the gene encoding Socs2 decreases after GH treatment in 3T3-F442A adipocytes as Socs2 expression rises [13]. Further, over-expression of Bcl6 inhibits Socs2-luciferase *in vitro* [13]. The present study provides evidence consistent with endogenous Bcl6 repressing Socs2 expression, since mesenchymal stem cells isolated from Bcl6-deficient mice exhibit a marked, cell autonomous increase in the expression of endogenous Socs2 mRNA. Additionally, Bcl6 KO mice exhibit increased expression of Socs2 mRNA in liver, adipose tissue, muscle and all other tissues examined.

In addition to stimulating Socs2 expression, GH is a potent inhibitor of Bcl6 expression; the latter suggests that the phenotype of Bcl6 KO mice in some ways reflects enhanced GH responses mediated by activation (de-repression) of GH target genes, such as those related to metabolic regulation. GH has long been known to regulate lipid metabolism by decreasing lipogenesis and increasing lipolysis [34], [35]. Mice overexpressing bovine GH (bGH), like Bcl6 KO mice, exhibit reduced epididymal adipose tissue relative to body weight [36]. The reduction of adipose tissue in bGH mice is not as dramatic as that observed in the absence of Bcl6, but does demonstrate a partial phenocopy of Bcl6 KO mice, and raises the possibility that changes in some functions of GH target genes on adipose tissue are involved. On the other hand, the elevated expression in Bcl6 KO mice of Socs2, a negative regulator of GH signaling [37], [38] suggests that Bcl6 KO mice might resemble a condition of Socs2 overexpression. Overexpressed Socs2 in porcine adipocytes has recently been reported to reduce expression of FAS and other genes involved in lipid metabolism [39], opening the possibility that Bcl6 dependent elevation of Socs2 in Bcl6 KO mice may contribute to some of the changes in lipid metabolism that were observed here. Recent reports indicate that deficiency of Socs2 in mice prevents hepatic steatosis with high fat diet, while it worsens insulin resistance, supporting metabolic roles for Socs2 [40]. Such relationships among Bcl6, Socs2, GH and metabolic genes do not appear to hold for the documented growth retardation of Bcl6 KO mice however [8], [41]. The growth regulatory events involving Socs2 and Bcl6 are not straightforward in the context of the GH-IGF axis, since transgenic mice overexpressing Socs2 are paradoxically larger than their wild type littermates [42], while Socs2 KO mice are also larger, consistent with Socs2 serving as a negative regulator of GH signaling. Further, on the Igf1 gene, Bcl6 does not appear to be a direct regulator of Igf1 expression, since Bcl6 occupancy on Igf1 was not regulated by GH [43].

### Bcl6 deficiency is associated with reduction of adipose tissue mass, hepatic triglycerides and lipogenic gene expression

Bcl6 has been reported to be expressed and regulated in adipocytes [13]. It is important to understand whether and how Bcl6 might participate in regulating adipocyte function, since the central role of adipocytes in metabolic regulation is well recognized under physiological and pathological conditions such as obesity and diabetes. The marked reduction of adipose tissue mass in Bcl6 KO mice, which can be considered a lipodystrophy, raises the idea that Bcl6 may contribute to adipogenesis, the process during which preadipocytes differentiate into metabolically functional, lipid-storing adipocytes. Numerous transcriptional activators and repressors orchestrate adipocyte differentiation [44]–[47]. A potential role for Bcl6 in adipogenesis is suggested by preliminary observations that Bcl6 mRNA increases during differentiation of 3T3-F442A preadipocytes to adipocytes (LaPensee and Schwartz, unpublished), though its adipogenic targets remain to be identified.

To address the metabolic consequences of Bcl6 deficiency in the face of the lack of adipose tissue in Bcl6 KO mice, metabolism was examined in liver, another crucial site of triglyceride synthesis and storage [48]. In addition to reduced adipose tissue mass, Bcl6 KO mice showed decreased levels of hepatic triglycerides and of genes encoding the enzymes FAS and SCD, which promote lipid synthesis. The dramatic reduction in expression of the rate-limiting lipogenic enzyme SCD1 may contribute to the phenotype of Bcl6 KO mice, as Scd1 knockout mice also exhibit reduced adipose tissue mass and decreases in Fasn mRNA expression [49].

Fasn and Scd1 gene expression in the liver is mediated by the transcription regulators ChREBP and SREBP1c [20], [27], [32], [50]–[52]. As observed in Bcl6 KO mice, where Chrebp expression is reduced, mice with total Chrebp deficiency exhibit reduced Fasn and Scd1 [32]. Further, adenoviral-mediated Chrebp knockdown in the liver of *ob/ob* mice causes a reduction in Fasn and Scd1 [18]. The finding that expression of the gene for GK, which is required for ChREBP regulation of lipogenic genes [27], is reduced in the liver of Bcl6 KO mice is consistent with a report that Chrebp and Fasn gene expression is decreased in hepatocytes from GK deficient mice [27]. The observation in Bcl6 KO mice of reduced Chrebp, Fasn and Scd1 expression despite unchanged Srebp1c mRNA levels is consistent with reports that ChREBP can mediate activation of lipogenic gene expression independently of SREBP1c [18].

Since Bcl6 KO mice exhibited a decrease in the expression of one enzyme for fatty acid oxidation, Acox, in conjunction with the decreased expression of lipogenic enzymes and reduced hepatic triglycerides, it is likely that reduction in pathways that promote lipid synthesis, rather than increased fatty acid oxidation, predominates in hepatic triglyceride metabolism during Bcl6 deficiency. Because Pparα RNA levels are comparable in WT and Bcl6 KO mice, the down-regulation of Acox observed in Bcl6 KO mice is most likely downstream of PPARα-induced gene transcription. One endogenous PPARα ligand is the FAS product 1-palmitoyl-2-oleoyl-*sn*-glycerol-3-phosphocholine (16:0/18:1-GPC), which induces expression of the PPARα-dependent genes Acox and Cpt1 [53]. Thus, low levels of hepatic FAS in Bcl6 KO mice might decrease availability of the PPARα ligand, subsequently lowering activation of PPARα and expression of Acox mRNA. Despite decreased expression of FA oxidation genes, Bcl6 deficient mice exhibit low triglyceride levels in liver, suggesting overall impairment in triglyceride metabolism during Bcl6 deficiency.

In addition to changes in gene expression, regulatory events involving post-translational modifications and cellular localization of key proteins of lipid metabolism such as SREBPs and Low Density Lipoprotein Receptor (LDLR) also play important roles in regulation of lipid metabolic pathways for triglyceride and cholesterol processing. For example, it is reasonable to speculate that activity of SREBP1c protein, which is dependent on its phosphorylation state, cleavage and re-localization in cells [31], [54] may be altered in the absence of Bcl6, and may also contribute to decreased activation of Fasn or Scd1 gene transcription, even when SREBP gene expression is not changed. SREBP proteins play multiple roles in lipid metabolism, including the regulation of cholesterol homeostasis as well as the lipogenic pathways explored here. SREBP-2 activates transcription of the gene encoding HMG-CoA reductase and other enzymes of cholesterol biosynthesis [55] LDLR participates in internalization of LDL for cholesterol metabolism via a pathway involving LDLR acidification in endosomes and receptor recycling [56]. If such modifications are altered in the absence of Bcl6, cholesterol metabolism may also be dysregulated. Future studies may reveal these and other features contributing to the lipodystrophy in Bcl6 KO mice.

### Bcl6 deficiency disrupts fasting-induced increase in hepatic triglyceride deposition

One could consider that reduced expression of hepatic Fasn and Scd1 observed in Bcl6 KO mice may be a consequence of reduced food intake, thus resembling fasting. However, several indicators make it unlikely that Bcl6 KO mice were nutritionally deprived. Non-fasted Bcl6 KO mice have blood glucose levels similar to those in WT mice, and blood glucose falls during fasting in KO mice to the same extent as in WT mice. If the phenotype of Bcl6 KO mice resembled a state of fasting, expression of Srebp1c, which decreases during fasting [19], [55], would be expected to be reduced. Instead, Srebp1c is expressed at similar levels in fed Bcl6 KO and WT mice, and fasting decreased Srebp1c expression to the same extent regardless of genotype. Consistent with the fasting-induced reduction in Srebp1c, expression of its target genes Fasn and Scd1 was also reduced further in both Bcl6 KO and WT mice during fasting, compared to fed mice of the same genotype [57].

Bcl6 deficiency disrupts the response to fasting by impairing the typical increase in liver triglycerides, but not the expected decrease in Fasn, Scd1, or Srebp1c mRNA. The inability of Bcl6 KO mice to increase hepatic triglycerides during fasting likely reflects at least in part the marked reduction in adipose tissue, and consequent reduction in the availability of adipocyte-derived fatty acids and glycerol for transport to the liver. Lack of hepatic triglyceride deposition is also reported in the liver of fasted Scd1 KO mice, suggesting that the dramatic reduction of Scd1 in Bcl6 KO mice is one of the underlying contributors to the low hepatic triglycerides during Bcl6 deficiency [49], [58].

### Multiple mechanisms of gene regulation by Bcl6

Because Bcl6 is known as a transcriptional repressor, Bcl6 deficiency would be expected to be associated with increased expression of genes directly inhibited by Bcl6, as demonstrated here for Socs2 [33]. However, expression of lipogenic genes Fasn, Scd1, and Chrebp was down-regulated rather than increased during Bcl6 deficiency, suggesting that these consequences of Bcl6 deficiency reflect mechanisms indirectly dependent on Bcl6.

Bcl6 represses genes that allow B-cell and T-cell differentiation and proliferation to proceed [1]–[3]. Given the crucial role of Bcl6 in the control of lymphocyte activation, differentiation, and apoptosis within the germinal center, it is reasonable to question whether the observed dysregulation of lipid metabolism is related or secondary to altered immune status in the absence of Bcl6. One indirect contributor to the consequences of the absence of Bcl6 may be the accumulation of T helper cell type 2 cells in peripheral lymphoid organs observed in Bcl6 KO mice [8]. Th2 cells produce interleukins, including IL-4, IL-5, and IL-13, which participate in the regulation of inflammatory and immune responses [59]. A set of preliminary observations appears to rule out IL-4 as a mediator of the changes in lipid metabolism in Bcl6 KO mice: When mice deficient in IL-4 alone (IL-4 KO) or in both Bcl6 and IL-4 (double KO) were compared, IL-4 KO mice were found to be identical to WT mice, and the double KO mice were identical to Bcl6 KO mice, with respect to fat mass and expression of Fasn, Scd1, and Chrebp and Srebp1c mRNA (LaPensee, Dent, Schwartz, unpublished). If IL-4 were a mediator of the changes associated with Bcl6 deficiency, one would have expected IL-4 KO mice to be comparable to Bcl6 KO rather than to WT, and for the double KO to reverse the consequences of Bcl6 KO to resemble WT rather than Bcl6 KO. Adding to this finding, mice with a targeted deletion of Bcl6 in T cells (CD4-cre) appear normal and do not exhibit growth retardation [60]. Similar findings of normal size and growth have been observed in mice with Bcl6 deleted in T regulatory cells (Foxp3-cre), B cells (CD19-cre), or macrophages (LysM-cre) (Dent, unpublished). Thus, some immune consequences of Bcl6 deficiency appear to be dissociated from dysregulated lipid metabolism and growth.

Examination of metabolic consequences of alterations of Bcl6 in human subjects may also provide insight into mechanisms relevant to lipid metabolism. The lack of clinical reports on the absence of Bcl6 makes it difficult to compare the present findings in Bcl6 KO mice to Bcl6 deficiency in humans. On the other hand, mutations and chromosomal translocations leading to Bcl6 over-expression is associated with about 40% of diffuse large B cell lymphomas [1], [3]–[5]. Altered lipid metabolism has been associated with B-cell lymphoma, although not directly linked to Bcl6 so far. For example, B-cells from some subsets of B-cell lymphoma are reported to show dysregulated fatty acid synthesis and glycolysis [61]. In mice with B-cell tumors induced by Bcr/Abl transformed B-cells, larger tumor size was associated with reduced adipose tissue mass, with the resulting mobilization of fatty acids supporting B-cell proliferation [62]. Thus metabolic consequences of B-cell metabolism may be related to the oncogenic properties of Bcl6. These observations are consistent with Bcl6 contributing to regulation of genes participating in metabolic regulation.

At the molecular level, the complex mechanisms by which a transcriptional regulator such as Bcl6 mediates changes in gene expression depend on its interactions and coordination with a host of other transcriptional regulatory proteins which associate with DNA of target genes [63]–[69]. The model of Bcl6 binding to regulatory sequence(s) of a gene to mediate repression may be relevant for a gene such as Socs2 in adipocytes and liver [11], [13], [43]. Activation of the Socs2 gene is mediated by Signal Transducer and Activator of Transcription (Stat) 5 in response to GH and other signals [70]. The Bcl6 consensus binding sequence is similar to that of Stat5, and Bcl6 occupies Socs2 at a functional Stat5 site. In fact, Bcl6 shares a consensus DNA binding sequence with Stat proteins [8]. Bcl6 and Stat5 are postulated to participate in an inverse transcriptional repressor/activator relationship [11], [13], [71], [72] which may be relevant for regulation of Socs2. Thus in Bcl6 deficiency, the increased expression of Socs2 likely reflects loss of repression by Bcl6, possibly in conjunction with activation by Stat5. For understanding of the role of Bcl6 in regulation of other genes, genome wide approaches such as chromatin immunoprecipitation coupled with deep sequencing are facilitating dissection of mechanisms by which Bcl6 regulates transcription, and can expedite identifying the changes in gene expression that mediate Bcl6 regulation of lipid metabolism suggested by these studies in Bcl6 deficient mice.

## Supporting Information

File S1 Fig. S1, Socs2 is elevated in multiple tissues of Bcl6-deficient mice. To determine whether Socs2 mRNA, which is elevated in adipose tissue, liver, and muscle, was also higher in kidney, spleen, and thymus, gene expression was compared by qpcr in tissues of male Bcl6 KO (black bars) and WT (grey bars) mice. mRNA expression is shown as the mean+SE for 4 mice of each genotype. Socs2 expression was significantly (p<0.05 *; p<0.005 **) elevated in Bcl6 KO mice in all tissues tested. Fig. S2, Socs2 is elevated in liver of female Bcl6-deficient mice. To determine whether Socs2 mRNA, which is high in male Bcl6 KO mice, is also elevated in female Bcl6 KO, Socs2 was measured in the liver of 5–6 week old female Bcl6 KO (black bar) and WT mice (grey bar) by qpcr. mRNA expression is shown as the mean+SE for 3 mice of each genotype. SOCS2 was significantly elevated (p<0.05 *) in liver of female Bcl6 KO mice. Fig. S3, Bcl6 KO mice are smaller than WT mice. A – Representative 6 week old male Bcl6 KO mouse is smaller than a WT littermate. B – Weights of male Bcl6 KO mice were significantly lower than WT littermates at 3, 4, and 5 weeks of age. Each point represents mean+SE for 4 WT (solid line) and 3 KO (dotted line) male mice. Asterisks (**) designate significant decrease (p<0.005) in Bcl6 KO relative to WT. Weights of female Bcl6 KO mice were similarly smaller (about 40%) than WT female littermates (data not shown). Fig. S4, Female Bcl6-deficient mice exhibit reduced hepatic triglycerides. To determine whether liver triglycerides (TG), which are low in male Bcl6 KO mice, are also reduced in female Bcl6 KO, triglycerides were isolated from the liver of 5–6 week old female Bcl6 KO and WT mice and were measured as described. Bars show the mean+SE (mg/gm tissue) for 3 mice of each genotype. Hepatic triglycerides were significantly lower (p<0.05) in Bcl6 KO relative to WT. Table S1.
(PDF)
