# Supplementary material for: Deficiency of the Transcriptional Repressor B Cell Lymphoma 6 (Bcl6) Is Accompanied by Dysregulated Lipid Metabolism
Source: PLoS One. 2014 Jun 3;9(6):e97090. doi: 10.1371/journal.pone.0097090 (PMC4043531; doi:10.1371/journal.pone.0097090)
Supplement: File S1 — Fig. S1, Socs2 is elevated in multiple tissues of Bcl6-deficient mice. To determine whether Socs2 mRNA, which is elevated in adipose tissue, liver, and muscle, was also higher in kidney, spleen, and thymus, gene expression was compared by qpcr in tissues of male Bcl6 KO (black bars) and WT (grey bars) mice. mRNA expression is shown as the mean+SE for 4 mice of each genotype. Socs2 expression was significantly (p<0.05 *; p<0.005 **) elevated in Bcl6 KO mice in all tissues tested. Fig. S2, Socs2 is elevated in liver of female Bcl6-deficient mice. To determine whether Socs2 mRNA, which is high in male Bcl6 KO mice, is also elevated in female Bcl6 KO, Socs2 was measured in the liver of 5–6 week old female Bcl6 KO (black bar) and WT mice (grey bar) by qpcr. mRNA expression is shown as the mean+SE for 3 mice of each genotype. SOCS2 was significantly elevated (p<0.05 *) in liver of female Bcl6 KO mice. Fig. S3, Bcl6 KO mice are smaller than WT mice. A – Representative 6 week old male Bcl6 KO mouse is smaller than a WT littermate. B – Weights of male Bcl6 KO mice were significantly lower than WT littermates at 3, 4, and 5 weeks of age. Each point represents mean+SE for 4 WT (solid line) and 3 KO (dotted line) male mice. Asterisks (**) designate significant decrease (p<0.005) in Bcl6 KO relative to WT. Weights of female Bcl6 KO mice were similarly smaller (about 40%) than WT female littermates (data not shown). Fig. S4, Female Bcl6-deficient mice exhibit reduced hepatic triglycerides. To determine whether liver triglycerides (TG), which are low in male Bcl6 KO mice, are also reduced in female Bcl6 KO, triglycerides were isolated from the liver of 5–6 week old female Bcl6 KO and WT mice and were measured as described. Bars show the mean+SE (mg/gm tissue) for 3 mice of each genotype. Hepatic triglycerides were significantly lower (p<0.05) in Bcl6 KO relative to WT. Table S1. (PDF) [file pone.0097090.s001.pdf]

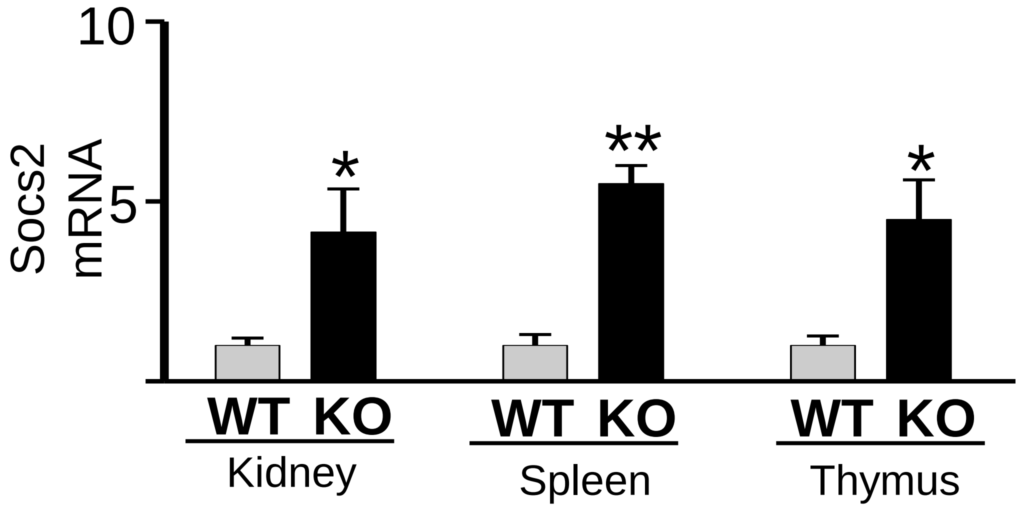

Fig S1. – Socs2 is elevated in multiple tissues of Bcl6-deficient mice. To determine whether Socs2 mRNA, which is elevated in adipose tissue, liver, and muscle, was also higher in kidney, spleen, and thymus, gene expression was compared by qpcr in tissues of male Bcl6 KO (black bars) and WT (grey bars) mice. mRNA expression is shown as the mean + SE for 4 mice of each genotype. Socs2 expression was significantly ($p<0.05$ *; $p<0.005$ **) elevated in Bcl6 KO mice in all tissues tested.

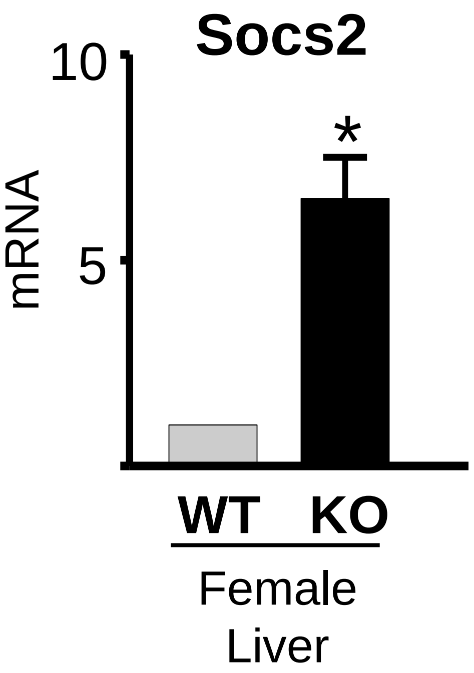

Fig S2. – Socs2 is elevated in liver of female Bcl6-deficient mice. To determine whether Socs2 mRNA, which is high in male Bcl6 KO mice, is also elevated in female Bcl6 KO, Socs2 was measured in the liver of 5-6 week old female Bcl6 KO (black bar) and WT mice (grey bar) by qpcr. mRNA expression is shown as the mean + SE for 3 mice of each genotype. SOCS2 was significantly elevated ($p<0.05$ *) in liver of female Bcl6 KO mice.

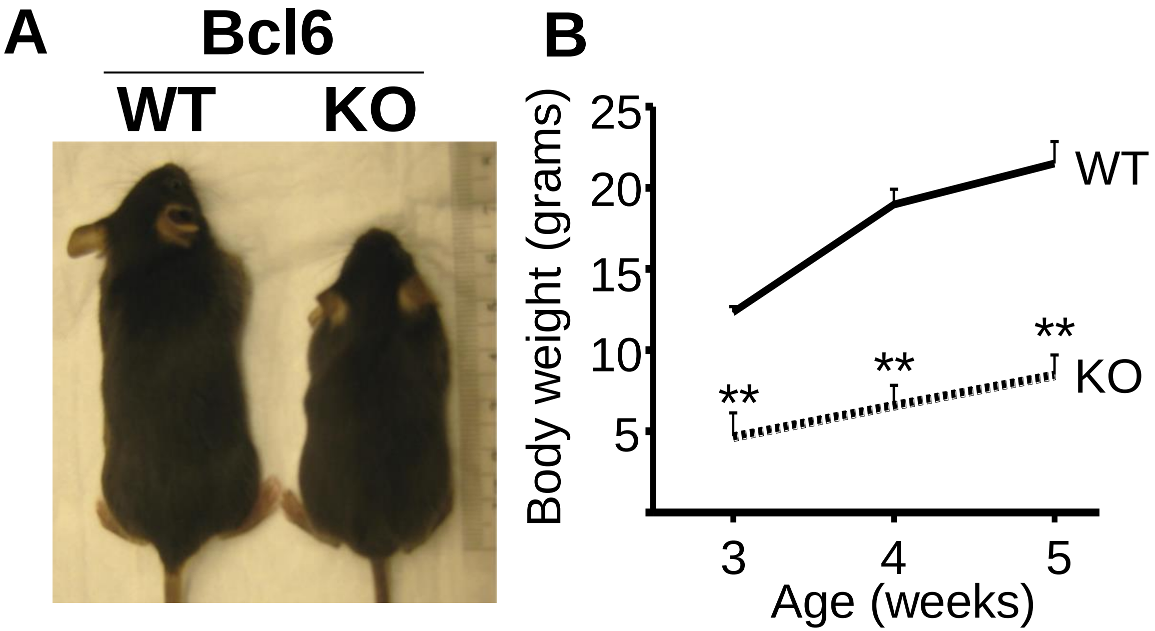

Fig S3. – Bcl6 KO mice are smaller than WT mice. A – Representative 6 week old male Bcl6 KO mouse is smaller than a WT littermate. **B** – Weights of male Bcl6 KO mice were significantly lower than WT littermates at 3, 4, and 5 weeks of age. Each point represents mean + SE for 4 WT (solid line) and 3 KO (dotted line) male mice. Asterisks (**) designate significant decrease (p<0.005) in Bcl6 KO relative to WT. Weights of female Bcl6 KO mice were similarly smaller (about 40%) than WT female littermates (data not shown).

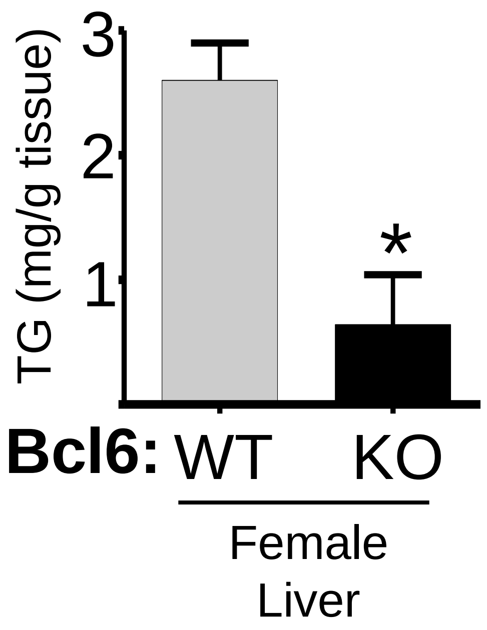

Fig S4. – Female Bcl6-deficient mice exhibit reduced hepatic triglycerides. To determine whether liver triglycerides (TG), which are low in male Bcl6 KO mice, are also reduced in female Bcl6 KO, triglycerides were isolated from the liver of 5-6 week old female Bcl6 KO and WT mice and were measured as described. Bars show the mean + SE (mg/gm tissue) for 3 mice of each genotype. Hepatic triglycerides were significantly lower ($p<0.05$) in Bcl6 KO relative to WT.

Supplementary Table S1

Gene	Forward Primer	Reverse Primer	Reference
Acox	ATATTTACGTCACGTTTACCCCGG	GGCAGGTCATTCAAGTACGACAC	(1)
Chrebp	GCATCCTCATCCGACCTTTA	GATGCTTGTGGAAGTGCTGA	(2)
Cpt1	GTCAAGCCAGACGAAGAACA	CGAGAAGACCTTGACCATAG	(3)
Fasn	TCCAGGCGCATGAGGCTCAGC	GGTTACACTGTCCTAGGTGTTG	(4)
GK	CCCTGAGTGGCTTACAGTTC	ACGGATGTGAGTGTTGAAGC	(5)
L-PK	CTTGCTCTACCGTGAGCCTC	ACCACAATCACCAGATCACC	(5)
Pparα	GGCTCGGAGGGCTCTGTCATC	ACATGCACTGGCAGCAGTGGA	(6)
Rplp0	GAAACTGCTGCCTCACATCCG	GCTGGCACAGTGACCTCACACG	(7)
Scd1	TGGGTTGGCTGCTTGTG	GCGTGGGCAGGATGAAG	(8)
Srebp1c	ATCGGCGCGGAAGCTGTCGGGGTA	ACTGTCTTGGTTGATGAGCTGGAGC	(9)
Socs2	TCTGGGGACTGCCTTTACCAAC	CCTCTGGGTTCTCTTTCACATAGC	(10)

References for Supplementary Table S1

1. **Uchida A, Slipchenko MN, Cheng JX, Buhman KK**. Fenofibrate, a peroxisome proliferator-activated receptor alpha agonist, alters triglyceride metabolism in enterocytes of mice. *Biochim Biophys Acta* 2011;1811:170-176.
2. **Wang RH, Li C, Deng CX**. Liver steatosis and increased ChREBP expression in mice carrying a liver specific SIRT1 null mutation under a normal feeding condition. *Int J Biol Sci* 2010;6:682-690.
3. **Kuhajda FP, Aja S, Tu Y, Han WF, Medghalchi SM, El Meskini R, Landree LE, Peterson JM, Daniels K, Wong K, Wydysh EA, Townsend CA, Ronnett GV**. Pharmacological glycerol-3-phosphate acyltransferase inhibition decreases food intake and adiposity and increases insulin sensitivity in diet-induced obesity. *Am J Physiol Regul Integr Comp Physiol* 2011;301:R116-130.
4. **Kennedy AR, Pissios P, Otu H, Roberson R, Xue B, Asakura K, Furukawa N, Marino FE, Liu FF, Kahn BB, Libermann TA, Maratos-Flier E**. A high-fat, ketogenic diet induces a unique metabolic state in mice. *Am J Physiol Endocrinol Metab* 2007;292:E1724-1739.
5. **Dentin R, Pegorier JP, Benhamed F, Foufelle F, Ferre P, Fauveau V, Magnuson MA, Girard J, Postic C**. Hepatic glucokinase is required for the synergistic action of ChREBP and SREBP-1c on glycolytic and lipogenic gene expression. *J Biol Chem* 2004;279:20314-20326.
6. **Jove M, Salla J, Planavila A, Cabrero A, Michalik L, Wahli W, Laguna JC, Vazquez-Carrera M**. Impaired expression of NADH dehydrogenase subunit 1 and PPARgamma coactivator-1 in skeletal muscle of ZDF rats: restoration by troglitazone. *J Lipid Res* 2004;45:113-123.
7. **Shi G, Zhang Z, Feng D, Xu Y, Lu Y, Wang J, Jiang J, Li X, Ning G**. Selection of reference genes for quantitative real-time reverse transcription-polymerase chain reaction in concanavalin A-induced hepatitis model. *Anal Biochem* 2010;401:81-90.
8. **Iankova I, Petersen RK, Annicotte JS, Chavey C, Hansen JB, Kratchmarova I, Sarruf D, Benkirane M, Kristiansen K, Fajas L**. Peroxisome proliferator-activated receptor gamma

recruits the positive transcription elongation factor b complex to activate transcription and promote adipogenesis. *Mol Endocrinol* 2006;20:1494-1505.

9. Primers for Srebp1c were designed using the NCBI Primer Blast tool (http://www.ncbi.nlm.nih.gov/tools/primer-blast/).

10. **Sugimoto N, Oida T, Hirota K, Nakamura K, Nomura T, Uchiyama T, Sakaguchi S**. Foxp3-dependent and -independent molecules specific for $CD25^{+}CD4^{+}$ natural regulatory T cells revealed by DNA microarray analysis. *Int. Immunol.* 2006;18:1197-1209.
